# Recombinant mistletoe lectin induces p53-independent apoptosis in tumour cells and cooperates with ionising radiation

**DOI:** 10.1038/sj.bjc.6600982

**Published:** 2003-05-27

**Authors:** K Hostanska, V Vuong, S Rocha, M S Soengas, C Glanzmann, R Saller, S Bodis, M Pruschy

**Affiliations:** 1 Department of Internal Medicine, University Hospital Zurich, Raemistr. 100, CH-8091 Zurich, Switzerland; 2 Department of Radiation Oncology, University Hospital Zurich, Raemistr. 100, CH-8091 Zurich, Switzerland; 3 Department of Dermatology, University of Michigan Health System, Ann Arbor, MI 48109, USA

**Keywords:** mistletoe lectin, apoptosis, p53, Apaf-1, ionising radiation

## Abstract

Mistletoe extracts are used as alternative cancer treatment in addition to standard chemotherapy and radiation treatment and have an immunostimulatory and pain-relieving effect. A direct antitumour effect of mistletoe extracts against tumour cells of lymphoid origin has been linked to the D-galactoside-specific mistletoe lectin I. In this study, we investigated the cellular effect of bacterially expressed, recombinant mistletoe lectin alone or in combination with ionising radiation in a genetically defined p53-wild-type and p53-deficient E1A/ras-transformed murine tumour cells system. Downregulation of the proliferative activity and cell killing by recombinant mistletoe lectin occurred in a clear dose response (0.1–1 ng ml^−1^). Induction of apoptosis was p53-independent, but apoptosis-associated factor-1-dependent. Cellular treatment with lectin in combination with ionising radiation resulted in both p53-wild-type and p53-deficient tumour cells in an at least additive, antiproliferative effect and enhanced activation of caspase-3. Combined treatment with ionising radiation and lectin revealed a similar cytotoxic effect in human, p53-mutated adenocarcinoma cells. Thus, recombinant mistletoe lectin alone and in combination with ionising radiation bypasses often prevalent apoptotic deficiencies in treatment-resistant tumour cells.

Mistletoe extracts are often used as complementary cancer treatment in addition to standard chemotherapy and radiation treatment and are reported to have an immunostimulatory and pain-relieving effect (Hajto *et al*, 1989; Beuth *et al*, 1992; Heiny *et al*, 1998; Stein and Berg, 1998; Ernst and Cassileth, 1999). A direct antitumour effect of mistletoe has been linked to the specific components lectin I–III that can induce apoptosis in various transformed cell lines, in particular in tumour cells of lymphoid origin. (Janssen *et al*, 1993; Büssing *et al*, 1996; Hostanska *et al*, 1996; Bantel *et al*, 1999). The main effector in mistletoe extracts presumably is the D-galactoside-specific mistletoe lectin I (ML-I). Mistletoe lectin-I is a heterodimer that consists of the toxic A-chain, a site-specific type II ribosome-inactivating *N*-glycosidase, and the carbohydrate-binding-subunit B responsible for cellular lectin uptake (Endo *et al*, 1988; Eck *et al*, 1999a,1999b).

Depending on the stimulus that initiates apoptosis, specific proteolytic caspase cascades, the core of the apoptotic programme, are activated. While death-ligand-mediated receptor activation facilitates the clustering and autoprocessing of caspases (initiator caspase-8) at the plasma membrane, other stress stimuli activate caspases at intracellular sites (Tepper *et al*, 1999). Mitochondrial cytochrome *c* is released into the cytosol upon cellular stress, where in the presence of ATP/dATP it associates with the apoptosis-associated factor 1 (Apaf-1) and procaspase-9 to process the protease to its active form (Liu *et al*, 1996; Li *et al*, 1997; Sun *et al*, 1999). This complex activates the effector caspase-3 leading to the observed cellular apoptotic morphology. Recently, induction of apoptosis in Jurkat leukemic T cells by ML-I was linked to activation of caspase-8 but independent of death receptor signalling (Bantel *et al*, 1999).

The state of the tumour suppressor p53 is pivotal for the response of tumour cells to chemo- and radiotherapy. Mutations in the p53 gene are involved in acquired and intrinsic treatment resistance in human tumours and render tumour cells refractory to many anticancer therapies (Lowe *et al*, 1994; Hainaut *et al*, 1998). Following irradiation, p53 is activated and induces a crucial block to cell cycle progression providing enough time for sufficient DNA-repair prior to deleterious DNA-replication in S phase. On the other hand, apoptosis may arise through p53-mediated signal transduction cascades leading to the activation of the apoptotic machinery. Various p53-inducible genes are known, although the specific apoptotic signalling network induced by p53 is only now emerging (Giaccia and Kastan, 1998; Soengas *et al*, 1999; Schuler *et al*, 2000). Thus, chemotherapeutic agents that alone or in combination with additional treatment modalities bypass the p53-dependent death pathway and induce p53-independent cell killing are interesting compounds for cancer treatment.

In this study, we investigated the cytotoxic effect and mechanism of cell death of recombinant ML alone or in combination with ionising radiation (IR) in a genetically defined p53-wild-type and p53-deficient tumour cell system and in cells lacking an intact Apaf-1/caspase-apoptotic pathway. This murine E1A/ras-transformed cell system has previously been described for its strict p53- or Apaf-1-dependent apoptotic response to treatment with IR and different cytotoxic drugs *in vitro* and *in vivo* (Lowe *et al*, 1993a; Soengas *et al*, 1999; Zaugg *et al*, 2001). Furthermore, the response to combined treatment has also been investigated in the human p53-mutated and IR-resistant colon adenocarcinoma cell line SW480.

## MATERIALS AND METHODS

### Recombinant ML and cell cultures

Recombinant ML (rML, rViscumin) was kindly provided by H Zinke (VISCUM GmbH, Zwingenberg, Germany). rML (*M*_r_ 55 kDa) was produced by cloning and separate expression of the two subunits A and B in *Escherichia coli* and renatured in a coassociation process (Eck *et al*, 1999a). E1A/T24 H-ras-transformed mouse embryo fibroblasts (MEFs) were cultured as described (Lowe *et al*, 1993b; Rocha *et al*, 2000). Unclonal mass cultures of E1A/ras-transformed Apaf-1-wild-type and deficient cells were prepared as previously described (Soengas *et al*, 1999) and cultured in Dulbecco's modified Eagle's medium 10% foetal calf serum (FCS), supplemented with penicillin and streptomycin. The human adenocarcinoma cell line SW480 was cultured in RPMI-1640-media supplemented with 10% FCS.

### Cell proliferation assay, trypan blue exclusion assay and irradiation

Treatment with rML was performed in presence of 2% FCS for 4 h followed by serum addition to 20% final serum concentration. For Annexin V-, Δ*ψ*_m_- and PI-exclusion-experiments (see below), the serum concentration was not readjusted. Control experiments were performed with the corresponding serum conditions. Tumour cell proliferation was assessed by the colorimetric alamar blue assay, a proliferation assay that assesses metabolic activity comparable to the MTT-tetrazolium-based [3-(4,5-dimethylthiazol-2-yl)2,5-diphenyltetrazolium bromide] quantification assay of cell metabolism (Biosource International, Camarillo, CA, USA). All proliferation experiments (in triplicate) were repeated as independent experiments at least twice. For trypan blue exclusion floating and adherent cells were collected, diluted 1 : 1 with 0.4% trypan blue solution (Sigma, Buchs, Switzerland) and scored under a light microscope.

The results represent the mean+s.d. of two independent experiments, with a minimum of 100 cells scored per treatment. Irradiation of cell cultures was carried out at room temperature in tissue culture dishes (100 × 100 mm) with a 6 MV linear accelerator at a dose rate of 2 Gy min^−1^ or in 96-well plates using a Pantak Therapax 300 kV X-ray unit at 0.7 Gy min^−1^ and was always applied 1 h following rML treatment.

### Analysis of cell viability, apoptotic nuclei, and Annexin V-binding by flow cytometry

Plasma membrane integrity was analysed by live–dead discrimination after staining with propidium iodide (PI, Sigma) at a final concentration of 5 *μ*g ml^−1^ for 15 min (Hostanska *et al*, 1996). The DNA content in treated cells was analysed by flow cytometry after staining in hypotonic fluorochrome solution (Nicoletti *et al*, 1991; Hostanska *et al*, 1996). Apoptotic cells were detected and quantified by staining with Annexin V-FITC (Roche Diagnostics, Rotkreuz, Switzerland) (Vermes *et al*, 1995) with a FACSCalibur flow cytometer (Becton Dickinson, Mountain View, CA, USA). The FACS histograms were analysed using CellQuest acquisition and analysis software.

### Mitochondrial transmembrane potential Δ*ψ*_m_

Cells (2 × 10^4^ per ml) were incubated with the cationic dye JC-1 (1 *μ*M, 5,5′, 6,6′-tetrachloro-1,1′,3,3′-tetraethylbenzimidazolocarbo-cyanine iodide, Molecular Probes, Eugene, USA) for 5 min at 37°C and analysed by flow cytometry (Zamzami *et al*, 1995). Changes from red (JC-1 aggregate) to green fluorescence (monomer) resulting from membrane depolarisation were evaluated on histograms. Statistical analysis of histograms was calculated using the Kolmogorov–Smirnov (K–S) two-sample test for overlaid histograms.

### Preparation of cytosolic cell fraction and Western blotting

Treatment with rML was performed in the presence of 2% FCS for 4 h followed by serum addition to 20% final serum concentration. Trypsin-treated cells were harvested and the cytosolic cell fraction was prepared as described previously (Rocha *et al*, 2000). Proteins were resolved by sodium dodecyl sulphate–polyacrylamide electrophoresis and Western blotting was performed with the primary rabbit polyclonal anti-cleaved caspase-3 antibody (New England Biolabs, Beverly, MA) and antibody detection was achieved by ECL-enhanced chemiluminescence (Amersham, Cardiff, UK) using a horseradish peroxidase-conjugated secondary antibody.

## RESULTS

### Cytotoxic effect of rML is p53-independent

**Figure 1:**
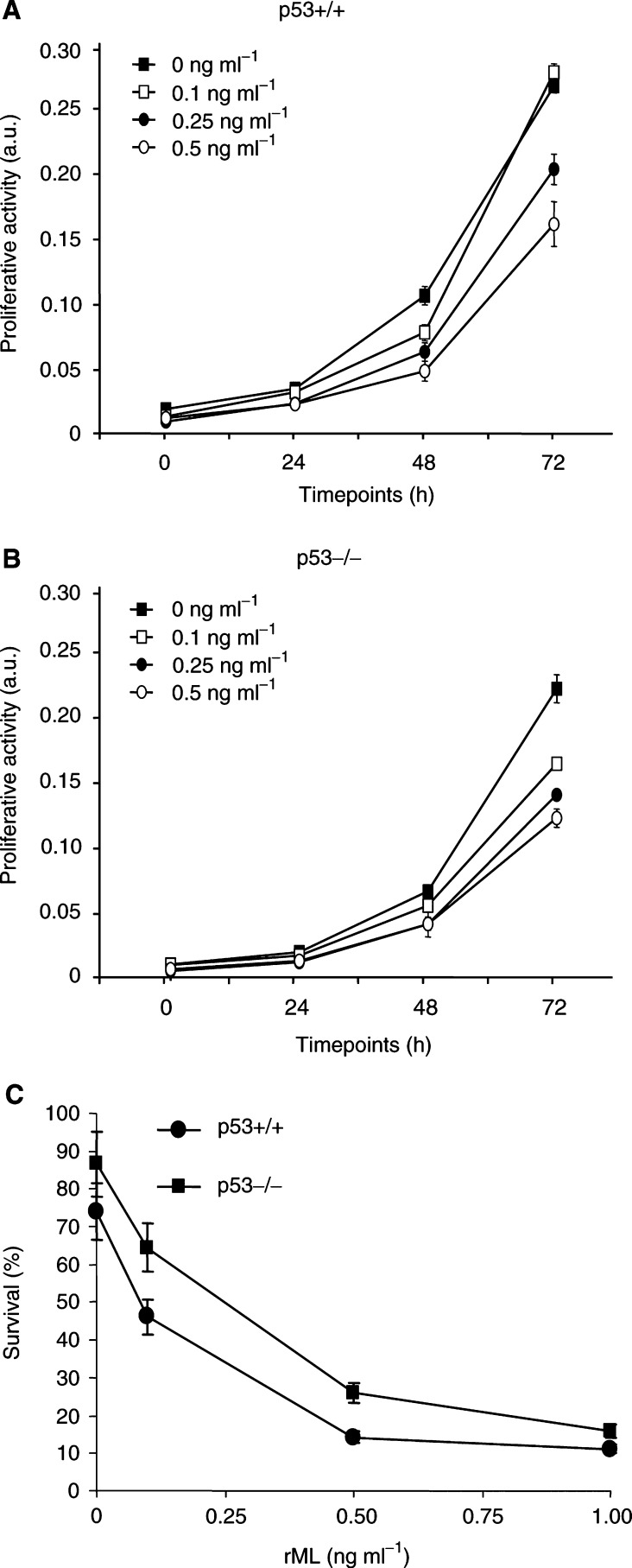
p53-independent antiproliferative and cytotoxic effect of rML. E1A/ras-transformed p53-wild-type (**A**) and p53-deficient (**B**) MEFs were treated with increasing concentrations of rML and proliferative activity was determined at the indicated timepoints with the alamar blue assay. (**C**) The cytotoxic effect was determined by PI exclusion 24 h after treatment with increasing concentrations of rML. The small amount of dead cells in p53-wild-type cells in the absence of treatment might be due to an increased rate of apoptosis induced by the short incubation time with low serum concentration (see Materials and Methods). Absence of error bars is due to minimal s.d.

The effect of rML on tumour cell proliferation was tested in E1A/ras-transformed p53-wild-type (+/+) and p53-deficient (−/−) murine embryo fibroblasts. This tumour cell system was previously described for its strict p53-dependent response to treatment with IR and different cytotoxic drugs *in vitro* and *in vivo* (Lowe *et al*, 1994; Pruschy *et al*, 1999; Rocha *et al*, 2000; Zaugg *et al*, 2001). To assess a p53-dependent stress response with these cells, initial control experiments were performed to determine the proliferative activity 48 h after serum withdrawal. When compared to the proliferative activity of cells growing in complete serum-supplemented medium, the proliferative activity of the p53-wild-type and p53-deficient cell population in the absence of serum were reduced to 2 and 39%, respectively, showing a clear p53-dependent stress response (data not shown). Treatment with increasing doses of rML (0–0.5 ng ml^−1^) drastically reduced the proliferative activity of both p53-wild-type and p53-deficient transformed cell populations. The proliferative activity was reduced in both cell populations in a clear dose response as tested over a 72 h time period after treatment (Figure 1A, B

). Treatment with higher doses of rML (1 ng ml^−1^) completely abrogated the metabolic activity (not shown). In parallel, the cytotoxic effect of rML-treatment in p53+/+ and p53−/− transformed cells was determined 24 h after treatment by PI exclusion. No significant difference in cell survival between these two cell lines was observed when cells were treated with increasing concentrations of rML (Figure 1C). Thus, the strongly reduced proliferative activity in the two cell populations by rML is most probably due to cell killing by rML, and these results indicate that rML induces its cytotoxic effect in a p53-independent way.

**Figure 2:**
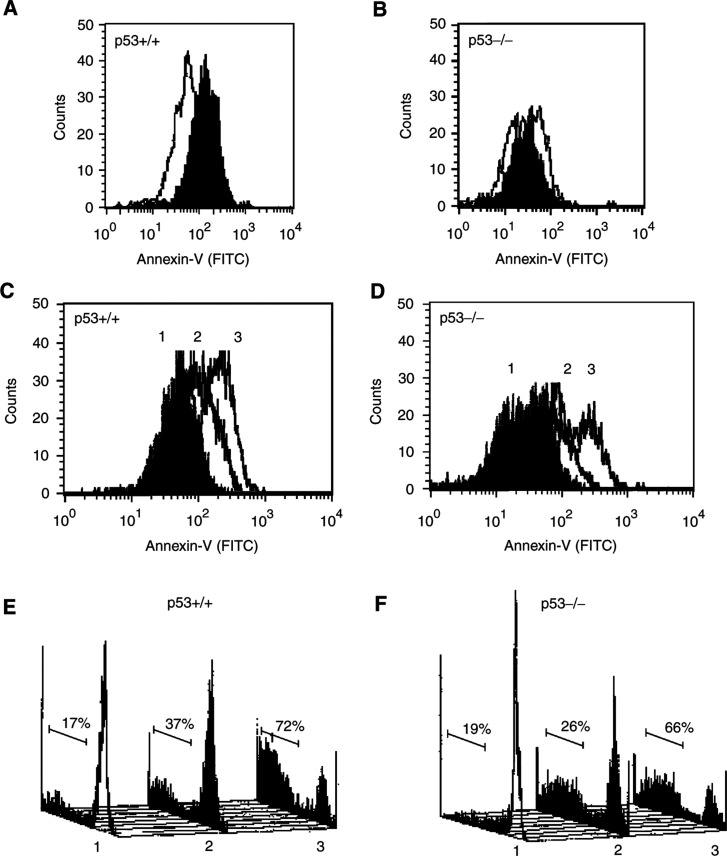
p53-independent induction of apoptosis by rML. Annexin V-binding was analysed by flow cytometry in E1A/ras-transformed p53-wild-type (**A**, **C**) and p53-deficient (**B**, **D**) MEFs 24 h after serum withdrawal (**A**, **B**, filled histograms) and after treatment with increasing concentrations of rML (**C**, **D**; 1: 0 ng ml^−1^ (filled histogram); 2: 0.1 ng ml^−1^; 3: 1 ng ml^−1^). (**E**, **F**) Apoptotic loss of DNA-content by flow cytometry of E1A/ras-transformed p53-wild-type (**E**) and p53-deficient (**F**) MEFs 24 h after treatment with rML (1: 0 ng ml^−1^; 2: 0.1 ng ml^−1^; 3: 1 ng ml^−1^). The *x*-axis represents the logarithmic scale of PI fluorescence intensity of nuclei and the *y*-axis the number of cells (the percentage of cells in the hypodiploid peak are indicated).

Next, the mode of cell death induced by rML-treatment was determined by Annexin V staining. As part of an apoptotic process, asymmetrically distributed plasma membrane phospholipids, for example, phosphatidylserine, become exposed on the outer cell membrane layer and this process can be assessed by Annexin V binding. Control experiments were performed to compare the response to rML with a strict p53-dependent induction of apoptosis (serum withdrawal) in these oncogene transformed cells. A large shift of Annexin V-binding-positive cells was detected 24 h after serum withdrawal in the p53+/+ but not in the p53−/− cell population (Figure 2A, B

; 77 *vs* 6% Annexin V-positive cells). Treatment with increasing concentrations of rML induced a dose- and time-dependent increase of Annexin V-positive staining cells. After treatment with 0.1 ng ml^−1^ rML for 4 h 10 and 3%, and after 24 h 63 and 29% of the p53+/+ and p53−/− cells, respectively, stained Annexin V positive. Treatment with an increased concentration of rML (1 ng ml^−1^) for 4 h resulted in 41 and 36% Annexin V-positive cells, and after 24 h 94 and 68% of the p53+/+ and p53−/− cells stained Annexin V positive (Figure 2C, D). In parallel, the DNA content in treated cells was analysed by flow cytometry. The apoptotic cells show a diminished DNA staining that can be traced below the G_0_/G_1_ population. A distinct hypodiploid (sub-G_1_) cell population was observed after treatment with increasing rML-doses. No significant differences in the amount of p53+/+ and p53−/− cells with apoptotic nuclei was observed 24 h after incubation with 0.1 ng ml^−1^ (37 and 26%, respectively) and 1 ng ml^−1^ rML (72 and 66%, respectively) (Figure 2E, F). These results indicate a predominantly p53-independent mechanism of apoptosis induction by rML.

**Figure 3:**
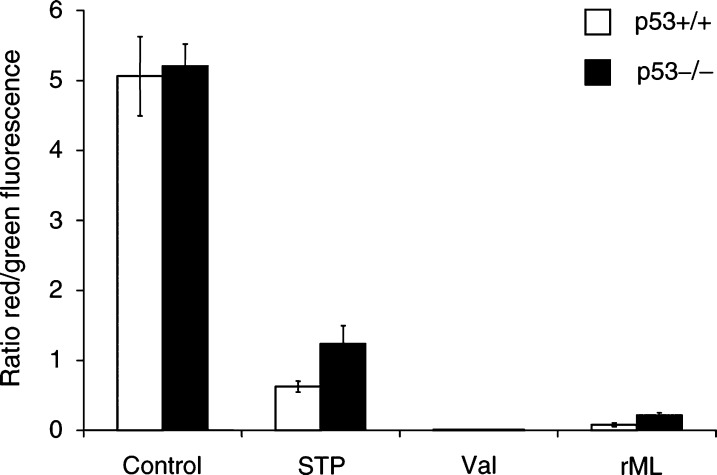
Loss of mitochondrial membrane potential by rML. The mitochondrial membrane potential of p53-wild-type and p53−/− deficient E1A/ras-transformed MEFs was analysed with JC-1 6 h after rML-treatment (0.5 ng ml^−1^). Control experiments were performed with valinomycin (50 nM, 15 min) and staurosporine (1 *μ*M, 3 h). The alterations from JC-1 aggregates (red fluorescence) to JC-1 monomer (green fluorescence) are presented as mean ratio (red/green fluorescence)+s.d. from two independent experiments.

A hallmark for apoptosis is loss or reduction of the mitochondrial membrane potential (Δ*Ψ*_m_). To investigate a change of Δ*Ψ*_m_ on rML-treatment, p53+/+ and p53−/− cells were incubated for 6 h with 0.5 ng ml^−1^ rML. As a positive control experiment, cells were treated for 15 min with the K^+^-ionophore valinomycin (50 nM) or for 3 h with staurosporine (1 *μ*M), which was previously shown to act as a p53-independent inducer of apoptosis (Rocha *et al*, 2000). 5,5′,6,6′-tetrachloro-1,1′,3,3′-tetraethyl-benzamida-zolocarbocyanin iodide (JC-1) is incorporated into mitochondria forming aggregates (red fluorescence) and monomers (green fluorescence), and mitochondrial depolarisation is indicated by a decrease in the ratio of red to green fluorescence. The ratio of red/green fluorescence in the untreated cell populations was 5.1 for the p53+/+ and 5.2 for the p53−/− cell population, respectively. As expected a complete loss of Δ*Ψ*_m_ occurred in both cell population upon addition of the uncoupler valinomycin (ratio 0.008). Likewise treatment of p53+/+ and p53−/− transformed cells with staurosporine and rML reduced Δ*Ψ*_m_ to a large extent in both cell population. Treatment with rML (0.5 ng ml^−1^) reduced the red/green-fluorescence ratio to 0.08 for the p53+/+ and to 0.22 for the p53−/− cell population showing p53-independent mitochondrial depolarisation by rML (Figure 3

). The corresponding ratio values after staurosporine-treatment were 0.62 and 1.24, respectively. Thus, mitochondrial depolarisation by staurosporine and rML correlate with their p53-independent cytotoxic activity.

Overall, these results obtained with a genetically defined tumour cell system indicate that the antiproliferative and cytotoxic effect of recombinant ML is due to p53-independent induction of apoptosis by rML.

### Apoptosis-associated factor-1-dependent induction of apoptosis by rML

**Figure 4:**
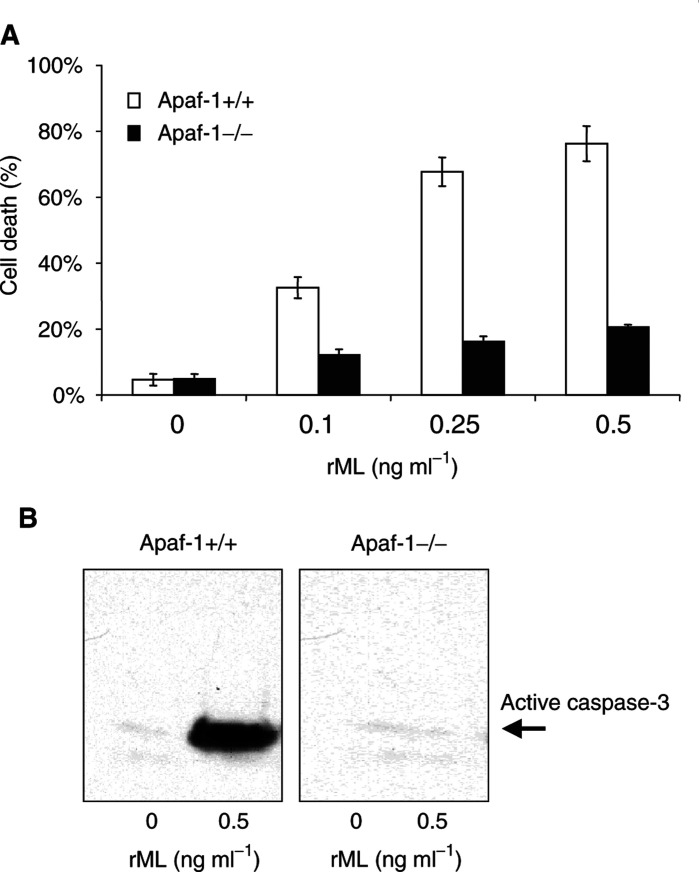
Apaf-1-dependent cytotoxicity and caspase-activation by rML-treatment. (**A**) Apaf-1-wild-type and Apaf-1-deficient MEFs were treated with rML (0–0.5 ng ml^−1^) and cell viability was determined by trypan blue exclusion 24 h after treatment. (**B**) Formation of active caspase-3 was determined in cytosolic extracts 18 h following treatment with rML using a specific antibody recognising the cleaved caspase-3 subunit.

To further explore the mechanism of rML-induced apoptosis, formation of caspase-3 was analysed in E1A/ras-transformed MEFs derived from Apaf-1-wild-type and Apaf-1-deficient mice. Apoptosis-associated factor-1 is an important cofactor for the assembly of the apoptosome complex that is required for activation of the initiator-caspase-9 and subsequent activation of caspase-3 as part of the cytochrome *c*-mediated caspase-9/-3 apoptotic pathway. The cytotoxic effect of increasing concentrations with rML (0–0.5 ng ml^−1^) was determined in Apaf-1+/+ and Apaf-1−/− E1A/ras-transformed MEFs. Quantitative analysis of dead cells was performed with the trypan blue exclusion assay. A dose-dependent increase of cell killing was observed in wild-type E1A/ras-transformed MEFs, while Apaf-1-deficient cells were strongly resistant to rML-treatment (77 *vs* 20% cell death at 0.5 ng ml^−1^ rML) (Figure 4A

). In parallel, cellular lysates derived from rML-treated E1A/ras-transformed Apaf-1+/+ and Apaf-1−/− cells were probed for active caspase-3-formation with the caspase-3-specific antibody. Interestingly, caspase-3-formation could only be detected in the wild type but not in the Apaf-1−/− cells (Figure 4B). These results indicate that rML requires an intact cytochrome *c*/Apaf-1/caspase-9-pathway for apoptosis induction.

### Combined effect by rML and irradiation

**Figure 5:**
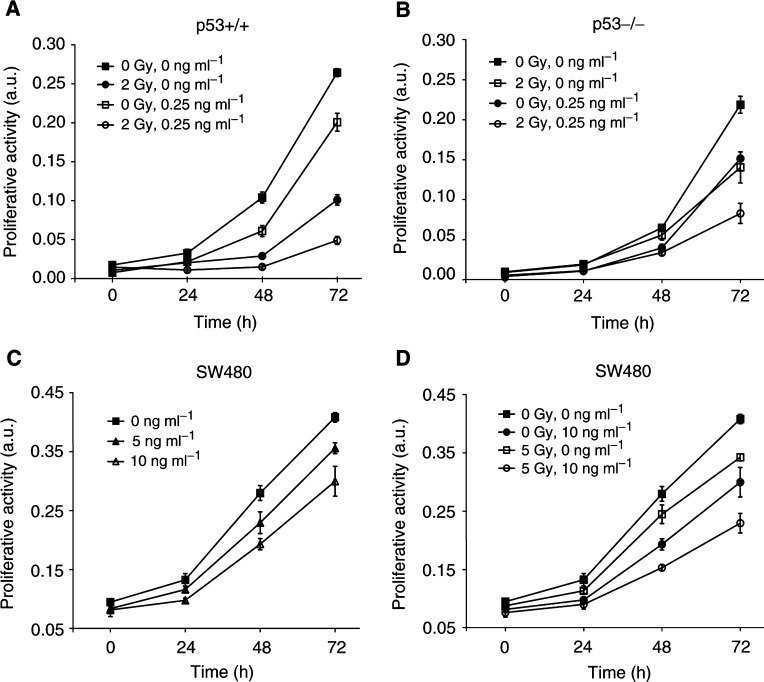
Combined antiproliferative effect by treatment with rML and IR. E1A/ras-transformed p53-wild-type (**A**) and p53-deficient MEFs (**B**) were treated with rML (0.25 ng ml^−1^) and IR (2 Gy) and the proliferative activity was assessed over 72 h after treatment with the MTT-like alamar blue assay. Irradiation was performed 1 h following rML treatment. The antiproliferative effect in the human SW480 cell line was also determined over 72 h after treatment with rML alone (**C**) or in combination with IR (**D**). Absence of error bars is due to minimal s.d.

Mistletoe extracts are often used as complementary cancer treatment in addition to standard radiation treatment, but the modulatory effect on treatment response is not clearly resolved. We tested the antiproliferative effect of combined treatment with low dose of rML and IR on the p53+/+ and p53−/− E1A/ras-transformed MEFs. As previously documented with this cell system, IR alone reduced the proliferative activity to a larger extent in the p53+/+ than in the p53−/− cells. Interestingly, combined treatment with rML (0.25 ng ml^−1^) and 2 Gy induced an at least additive antiproliferative effect in both cell populations. Experiments to determine the antiproliferative effect of rML and IR were also performed against the human p53-mutated adenocarcinoma cell line SW480, which has previously shown to be rather radiation resistant (Hess *et al*, 2001). Treatment with increasing doses of rML alone reduced the proliferative activity in a dose-dependent way and also showed an at least additive effect when tested in combination with IR (Figure 5A–D

). Trypan blue exclusion performed on SW480 cells corroborated that the lectin alone has only a partial effect on cell viability, while IR-induced cell killing was increased in a cooperative way by rML in these cells (not shown).

**Figure 6:**
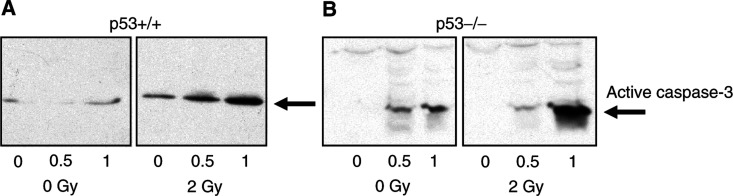
p53-independent caspase-3-activation upon combined treatment with rML and IR. E1A/ras-transformed p53-wild-type (**A**) and p53-deficient (**B**) MEFs were treated with rML (0, 0.5, 1 ng ml^−1^) alone or in combination with irradiation (2 Gy). Irradiation was performed 1 h after rML treatment and formation of active caspase-3 was determined in cytosolic extracts 18 h following irradiation using a specific antibody recognising the cleaved caspase-3 subunit.

Formation of active effector-caspase-3 upon treatment with rML alone and in combination with IR was determined in cytosolic lysates derived from the genetically defined p53+/+ and p53−/− cells by Western blotting to further understand the combined effect on the molecular level. A caspase-3-specific antibody was used that recognises only the cleaved, active form of caspase-3 (Figure 6A, B

). Treatment with increasing concentrations of rML (0, 0.5, 1 ng ml^−1^) activated caspase-3 in both cell population, supporting p53-independent apoptosis-induction by rML as observed on the cellular level (see above). When rML-treatment was combined with IR (2 Gy) formation of the active form of caspase-3 was at least additive in both p53+/+ and p53−/− cell population, suggesting that the increased antiproliferative effect by rML in combination with IR is because of enhanced apoptosis-induction.

## DISCUSSION

This report investigates the antiproliferative and cytotoxic effect of rML alone and in combination with IR in a murine genetically defined p53+/+ and p53−/− tumour cell system, which has been previously used to determine p53-dependent apoptosis induction (Lowe *et al*, 1993b,^1994^), and in human p53-mutated colon adenocarcinoma cells. Both p53-wild-type and p53-inactive cell types were sensitive to increasing concentrations of rML. Furthermore, mechanistic investigations with the genetically defined tumour cell system show that breakdown of the mitochondrial membrane potential and caspase-3-activation occurred in a p53-independent way upon treatment by rML. More important, the p53-independent, antiproliferative effect by recombinant ML well correlated with recombinant ML-induced cell killing irrespective of the p53-status. Interestingly, p53-independent apoptosis-induction was also recently demonstrated in different tumour cells with a related lectin purified from Korean mistletoe (Lyu *et al*, 2002).

Apoptosis was also enhanced in both cell types when rML was used in combination with IR, which induces apoptosis in a strict p53-dependent way when applied as single treatment modality. rML-induced apoptosis, though, was abrogated in cells lacking an intact cytochrome *c*-mediated apoptotic pathway, suggesting that rML acts downstream of p53 but upstream of the apoptotic caspase machinery.

In tumour cells of lymphoid origin, it was previously demonstrated that ML-I induces the apoptotic machinery in a cell death-receptor-independent way and that ML-I and the Korean mistletoe ML-II activated both initiator caspases-8 and -9 (Bantel *et al*, 1999; Kim *et al*, 2000). Using a genetically defined system for Apaf-1, we show here that rML-induced apoptosis leading to caspase-3-activation and rML-mediated cytotoxicity not only activates but strictly requires this apoptosome-dependent-pathway. In Apaf-1-deficient E1A/ras-deficient cells, rML-1 did not activate effector caspase-3 and these cells were resistant to the cytotoxic effect of rML.

Only limited information is present on the initial mechanism of apoptosis induction by MLs. Mistletoe lectins consist of a toxic A-chain, the site-specific ribosome-inactivating *N*-glycosidase, and a carbohydrate-binding-subunit B. The B-chain is important for cellular lectin uptake, internalisation of the A-chain and is partially involved in the induction of cytokine synthesis (Hajto *et al*, 1990), but does not induce apoptosis on its own (Hostanska *et al*, 1996; Eck *et al*, 1999b). On the other hand, the cytotoxic and apoptosis-inducing effect of ML is exerted by the ribosome inactivating A-chain (Hostanska *et al*, 1996; Langer *et al*, 1999). Most probably it is this protein synthesis-inhibiting subunit that is responsible for the p53-independent apoptosis-inducing and cytotoxic effect of rML. Controlled protein synthesis is crucial for the survival of any cell and the coordinated induction of apoptosis may be linked to the downregulation of short-lived proteins that are important for cellular homeostasis and a high apoptotic threshold. The observed cooperative effect of rML-treatment when used in combination with IR might be due to the enhancement of this antihomeostatic response to rML. Combined treatment resulted in an increased amount of caspase-3-activation in both p53+/+ and p53−/− cells indicating that the cooperative IR-effect is not mediated via p53 either. However, which proteins are immediately affected by rML alone and in combination with IR and their relation to the apoptotic machinery is not known so far.

p53-independent interference of gene expression and protein synthesis resulting in apoptosis is also known for other protein synthesis inhibitors or cytotoxins such as onconase, and Pseudomonas exotoxin. Likewise ricin, which also belongs to the class of two-chain (type II) ribosome-inactivating proteins, induces apoptosis in p53-mutated tumour cells (Wu *et al*, 1993; Rodriguez *et al*, 1998). This is in contrast to other stress factors that also affect the cellular homeostasis such as hypoxia, oncogene activation or aberrant nucleotide metabolism that induce apoptosis although in a p53-dependent way (Giaccia and Kastan, 1998).

The antiproliferative and cytotoxic effect of rML alone and in combination with IR was prominent both in the murine genetically defined, oncogene-transformed tumour cell system and in the human p53-mutated colon adenocarcinoma cell line. Even though the potential antitumour effect of mistletoe extracts has been linked to its immunomodulatory mechanism mediated by natural killer cells, lymphokine-activated killer cells and macrophages, a cytotoxic effect of purified protein components directed against tumour cells has been previously reported *in vitro* and *in vivo* (Hajto *et al*, 1989,^1998^; Beuth *et al*, 1991; Yoon *et al*, 1995,^1998^; Baxevanis *et al*, 1998; Schumacher *et al*, 2000; Elsasser-Beile *et al*, 2001). The cytotoxic characteristics described for the recombinant ML in this report will not necessarily predict the cytotoxicity profile of mistletoe extract or lectins purified from extracts, even though the immunomodulatory and apoptosis-inducing properties of the extract correlate with their content of lectins (Haito *et al*, 1998; Büssing and Schietzel, 1999; Hostanska *et al*, 1999). For example, minor extract components like viscotoxins, a group of 5 kDa polypeptides, might modify the tolerability, specificity and properties of standardised lectin extracts (Tabiasco *et al*, 2002). The biological concept of ML-induced cytotoxicity is intriguing. This report demonstrates that a recombinant component of mistletoe extracts overcomes a high apoptotic threshold and cooperates with IR in tumour cells that lack intact p53 and that are resistant against classical chemotherapeutics. Thus, it will be important to identify the specific molecular sensor that triggers p53-independent induction of the apoptotic machinery by rML.
